# Complete Genome Sequence of Celeribacter baekdonensis Strain LH4, a Thiosulfate-Oxidizing Alphaproteobacterial Isolate from Gulf of Mexico Continental Slope Sediments

**DOI:** 10.1128/genomeA.00434-18

**Published:** 2018-05-17

**Authors:** Beverly E. Flood, Dalton Leprich, Jake V. Bailey

**Affiliations:** a Department of Earth Sciences, University of Minnesota, Minneapolis, Minnesota, USA

## Abstract

We report here the closed genome sequences of Celeribacter baekdonensis strain LH4 and five unnamed plasmids obtained through PacBio sequencing with 99.99% consensus concordance. The genomes contained several distinctive features not found in other published Celeribacter genomes, including the potential to aerobically degrade styrene and other phenolic compounds.

## GENOME ANNOUNCEMENT

The genus Celeribacter is a recently described clade within the roseobacterial group (1) for which there are currently nine isolates and eight genomes. All nine isolates are heterotrophic marine aerobes that lack carotenoids and bacteriochlorophyll *a* (2). Notable reported metabolisms include manganese oxidation (3), polycyclic aromatic hydrocarbon (PAH) degradation (4–7), ethanol degradation (8), and acid production via the degradation of sugars and polyols (9). We isolated strain LH4, previously referred to as Roseobacter strain LH4, from sediments surrounding a brine pool in the Green Canyon Block 233 on the mid-continental slope, Gulf of Mexico (27°43.4392ʹN, 91°16.7638ʹW; depth, 648 m). Strain LH4 oxidized thiosulfate-producing acid in the presence of barium, and encrusting barite precipitates formed (10). The agar medium was designed to promote chemolithoautotrophic thiosulfate oxidation. An additional observation included biofilm formation occurring on mineral surfaces.

More than 14 µg of DNA from a batch culture grown aerobically on modified marine broth 2216 was extracted and purified using a Qiagen Genomic-tip 20/G. Size selection with a 3-kb cutoff was performed to obtain ∼20-kb long reads using BluePippin technology (Sage Science). The reads were then sequenced via 120-min movies in 2 single-molecule real-time (SRMT) cells using P6-C2 chemistry on a PacBio RS II sequencer (Mayo Clinic Bioinformatics Core, Rochester, MN). The genome filtering and assembly were performed using tools within SMRT Analysis version 2.1 (11). Raw reads were filtered to remove SMRTbell adapters and short and low-quality reads. *De novo* assembly was performed via HGAP 3.0 on self-corrected long reads, with a minimum length cutoff of 10,000 bp, resulting in ∼215× coverage of the bacterial chromosome and between 102 and 217× coverage of the plasmid genomes. The final genome of strain LH4 was 3,586,885 bp (G+C content, 58%). The final genomes of the plasmids were 420,660 bp (G+C content, 58%), 123,520 bp (G+C content, 60%), 127,557 bp (G+C content, 60%), 64,140 bp (G+C content, 58%), and 114,606 bp (60%). Annotation was performed using the NCBI Prokaryote Genomes Annotation Pipeline and the JGI Integrated Microbial Genomes Pipeline (12). Protein-coding genes with a predicted function comprised 76.05% of the genome and plasmids.

The genome sequence of Celeribacter baekdonensis strain LH4 indicates that it is not autotrophic and thus likely scavenges trace organics from agar to facilitate growth. The observed acid-producing thiosulfate oxidation pathway is likely the *sox* pathway (*soxABCDXYZ*). Other genes related to sulfur oxidation include four that code for sulfide quinone oxidoreductases and three for flavocytochrome *c*; both of these enzymes are known for the oxidation of H_2_S. Notable attributes in the genome of strain LH4 include traits observed in some celeribacters, such as mixed acid fermentation to H_2_ and CO_2_ (formate hydrogen lyase) and the capacity to degrade PAHs via plasmid-carried genes (*paaABCDEGHJKXYZ* ). Unique to strain LH4 are plasmid-carried genes for styrene degradation, styrene monooxygenase (*styA*), and styrene-oxide isomerase (*styC*). Strain LH4 also has phage-encoding genes between bases 441220 to 543983 in its chromosome.

### Accession number(s).

The assembly and annotation files were deposited in GenBank under the accession numbers CP028472 to CP028477. The annotated genome is also available through the Integrated Microbial Genomes database under taxon identification (ID) 2737471647.
